# Socio-economic analysis in REACH restriction dossiers for chemicals management: A critical review

**DOI:** 10.1007/s13280-019-01285-9

**Published:** 2019-12-05

**Authors:** Silke Gabbert, Isabel Hilber

**Affiliations:** 1 Department of Social Sciences, Environmental Economics and Natural Resources Group, Wageningen University & Research, Hollandseweg 1, 6700 EW Wageningen, The Netherlands; 2 Agroscope, Reckenholzstrasse 191, 8046 Zurich, Switzerland

**Keywords:** Impact assessment, REACH, Regulation, Restriction of chemicals, Risk management, Socio-economic analysis

## Abstract

This paper offers a critical review of socio-economic analysis (SEA) in Registration, Evaluation, Authorisation and Restriction of Chemicals (REACH) restriction dossiers. We examine the conceptual setup of SEA and identify the methods used for impact assessment. Moreover, we analyse the outcomes of quantitative impact assessment across restriction dossiers and substance groups. We find that impact assessment has largely focused on economic and health impacts. Environmental, social, wider economic and distributional impacts have either been evaluated qualitatively or not at all. While this can be explained by the specific scope of the proposed restriction or by lacking data, we also observe a lack of approaches for environmental and health impact assessment. This underlines the need to develop integrated methods that transform information about chemical effects and risks into impacts and, ultimately, into benefits and damages. Furthermore, to strengthen the function of SEA as decision-support tool in REACH restriction procedures, a comparative SEA of at least two alternative restriction options should be the default.

**Electronic supplementary material:**

The online version of this article (10.1007/s13280-019-01285-9) contains supplementary material, which is available to authorized users.

## Introduction

The ambition of the European chemicals’ legislation REACH (Registration, Evaluation, Authorisation and Restriction of Chemicals) is to ensure “a high level of protection of human health and the environment, including promoting alternative methods for assessment of hazards of substances, while enhancing competitiveness and innovation” (European Commission 2006). There is wide agreement that REACH has significantly improved information about the properties and the risks of chemicals in use, or intended to be placed on the market in amounts larger than 1 tonne/year (Hansen et al. 2007; Getzner and Schulz-Zak 2018). Furthermore, reversing the ‘burden of proof’, i.e. shifting the responsibility to provide relevant information about chemical properties, exposures and their (safe) use(s) from regulatory authorities to chemical producers, manufacturers and downstream users, has been considered a paradigm-change in European chemicals risk management (Hansen et al. 2007; Béal and Deschamps 2016). While a comprehensive, quantitative assessment of the short- and long-term impacts of REACH at the European level has not become available as yet, several studies have presented estimates of the expected environmental, clean-up, public and occupational health costs saved (cf. Reihlen and Lüskow 2007 for a survey).

REACH adopts two complementary regulatory instruments, (i) authorisation, i.e. the formal approval to continue one or multiple uses of a substance for a defined time period, and (ii) restriction, i.e. the enforcement of measures to reduce or eliminate the risks from producing, manufacturing and marketing a substance. An authorisation process is initiated by companies, i.e. producers, manufacturers and downstream users, who can apply for authorisation of specific uses of ‘Substances of Very High Concern” (SVHC, European Chemicals Agency (ECHA) 2019a and European Commission (EC) 2006, Title VII, see also Klika 2015), pure or in a mixture. SVHCs are chemicals which are classified as one of the following (i) carcinogenic, mutagenic or toxic for reproduction, (ii) persistent, bioaccumulative or toxic (PBT) or very persistent and very bioaccumulative (vPvB), (iii) endocrine disrupters, or substances that (iv) give “rise to an equivalent level of concern” (EC 2006, Article 57). A restriction process, by contrast, can either be initiated by regulatory authorities of EU member states or by the ECHA on request of the EC. Restrictions apply to any substance, to substances in a mixture or in a product. Restricted substances can, but do not have to be SVHC (ECHA 2019b). For both authorisation and restriction procedures, the EC takes the final decision, taking into account the written opinions of the ECHA Risk Assessment Committee (RAC) and the ECHA Socio-economic Assessment Committee (SEAC). Whereas a decision on authorisation is binding for the authorisation holder only, a decision on restriction applies to all companies (i.e. producers, manufacturers, downstream users, retailers) of EU member states. Regulatory authorities of EU member states are responsible for its enforcement (ECHA 2007, 2019b).

The focus of this paper is on REACH restrictions. Most commonly, a restriction process is initiated when an EU member state, or ECHA on request of the EC, starts preparing an Annex XV restriction dossier (ECHA 2019e). Based on the written opinions of RAC and SEAC, the applicant prepares a final background document of the proposed restriction. A restriction dossier includes detailed information about (i) the suggested restriction of a substance’s use, (ii) its hazards, risks, and existing alternatives (e.g. the use of substitutes or alternative technologies), (iii) the need to adopt regulatory measures at an EU-wide level, and (iv) the expected positive and negative impacts of the proposed restriction or other possible risk management options (RMOs). Finally, a restriction dossier reports (v) results of stakeholder consultations regarding possible impacts of the proposed restriction.

For assessing expected impacts, dossier submitters can add a socio-economic analysis (SEA). Generally, an SEA is “an approach to analysing all relevant impacts (i.e. both positive and negative changes) of one scenario against another“, and serves as “a decision-support tool to evaluate the costs and benefits an action will create for society by comparing what will happen if this action is implemented as compared to the situation where the action is not implemented” (ECHA 2008, 2011). In the REACH restriction process, an SEA can be used in order to motivate the need for community-wide action, to justify the appropriateness of a restriction proposal, or to assess the expected benefits and costs for different societal actors (manufacturers, importers, consumers, etc.) under the proposed restriction (ECHA 2008). Though an SEA is not mandatory for REACH restrictions, it is recommended to be included into a restriction proposal in order to assess the proportionality, i.e. the societal acceptability, of the proposed restriction (ECHA 2008). Consequently, most restriction dossiers contain an SEA (ECHA 2019d), which underlines its perceived strategic relevance for decision-making. To facilitate SEA in REACH restriction procedures, the ECHA has published guidance documents outlining the different components of an SEA and possible methodological approaches (ECHA 2008, 2010). So far, however, there has been little insight on how SEAs have been applied in REACH restriction procedures.

Conducting an SEA is a time- and resource-consuming task. An obvious and relevant question is, therefore, how applicants have accomplished SEAs in REACH restriction dossiers, and what outcomes have become available from the assessments. This paper offers a systematic and structured evaluation of SEAs in 24 REACH restriction dossiers. Specifically, we explore (i) the purpose(s) of an SEA, (ii) the types of impacts assessed, (iii) the methodological approaches used for impact assessment and aggregation, and (iv) the main quantitative outcomes of an SEA. Particular attention is given to comparing results between substances classified as SVHC and non-SVHC. Under the REACH legislation, SVHCs are usually aimed to be phased-out unless the EC has granted a formal authorisation for specific uses of the substance. A restriction applying to all uses and EU member states can be more efficient and easier to implement than a ban on specific uses only. However, if the substance has a widespread use and cannot straightforwardly be substituted by an alternative substance or technology, EU member states may have a strong incentive to restrict an SVHC. Based on our findings regarding (i)–(iv), we discuss (v) the contribution of an SEA for decision-making on restrictions.

Closest to our study is a recent report published by the ECHA, which compares aggregate cost and benefit information retrieved from 18 adopted restriction proposals (ECHA 2016). The report documents cost and benefit estimates that are presented in the final background documents for the chosen restriction. Our paper goes beyond this analysis by addressing a broader sample of restriction dossiers and by providing an in-depth analysis of the methodological approaches adopted in and the outcomes of SEAs.

The remainder of the paper is organised as follows: The “Materials and Methods” section informs about the materials and methods used. Specifically, we discuss the approach adopted for the comparative evaluation of SEA based on defined plausibility criteria. Following to this, we discuss the practical implementation of SEA in REACH restriction dossiers. The “Discussion” section concludes and points to implications from our analysis for decision-making on restrictions under REACH.

## Materials and Methods

### Restriction dossiers included in the evaluation

So far, restrictions have been adopted for 70 chemicals (ECHA 2019d). Of these, 45 substances had already been restricted under the previous legislation, EU Directive 76/769/EEC (EU 1976). These restrictions were formally converted to REACH restrictions without passing through the regular procedure of submitting a REACH Annex XV restriction dossier (ECHA 2019d). For 25 substances, restriction procedures were conducted according to REACH guidelines (ECHA 2007). For 24 of these, (draft) opinions from the ECHA Risk Assessment and the Socio-economic Assessment Committee (RAC and SEAC) have been adopted until December 2018 and were included in our analysis.

Detailed documentation about the different stages of the restriction process per substance (i.e. restriction proposal, draft and final RAC and SEAC opinions including minority positions, submitters’ responses to RAC/SEAC opinions, final background document, adopted restriction) is available on the ECHA website (https://www.echa.europa.eu/substances-restricted-under-reach). For the comparative evaluation of SEA, we used the final background documents, i.e. the restriction proposals amended by comments based on the opinions of the RAC and the SEAC (ECHA 2019d). Table S1 in the Electronic Supplementary Material (ESM) provides basic information about the dossiers included in the analysis, i.e. the substance names and their CAS numbers, the date of dossier submission, and the name of the submitting authority. The table also indicates whether a substance belongs to the group of SVHC according to REACH criteria. Furthermore, the table presents the baseline scenario for SEA, the RMOs specified by the dossier submitter and, where available, the regulatory decision adopted by the EC.

### The scope of SEA in REACH restriction dossiers

**Fig. 1:**
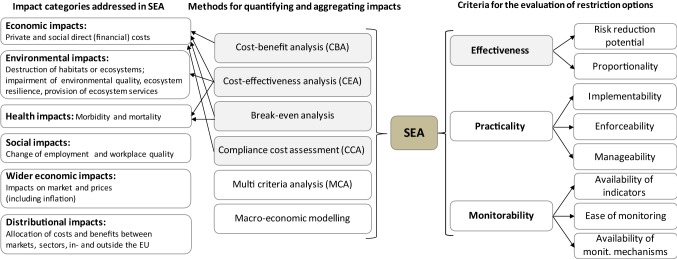
Impact categories addressed in SEA for REACH restriction procedures, methods suggested for quantifying and aggregating impacts in SEA, methods used in SEA (grey-shaded), criteria for evaluating restriction options, and evaluation criteria that were informed by SEA (grey-shaded). Source: Adapted from ECHA (2008). Arrows indicate the methods that were applied for assessing an impact category

A REACH Annex XV restriction dossier usually starts with formulating a ‘proposed restriction’, i.e. a control measure to reduce or eliminate the risks arising from the use of a substance on its own or in articles. This can be a control measure prohibiting manufacture or the use of a substance in articles, their placement on the market, occupational safety measures, a market removal of products containing a substance, the implementation of new technology, or the establishment of upper concentration limits for a substance's use in articles. Besides the proposed restriction, the dossier submitter should identify possible alternative RMOs, including measures under other existing Community legislation (e.g. the European Water Framework Directive, EC 2000), existing sector- and use-specific legislation, or voluntary risk management measures to be adopted by industry. The dossier submitter must motivate why the proposed restriction, or any of the alternative RMOs, is the most appropriate control measure on a Community-wide basis. This is done by assessing an RMO’s effectiveness, practicality, and monitorability (ECHA 2008). These base criteria, in turn, consist of different sub-criteria as shown in Fig. 1. ‘Effectiveness’ addresses the expected risk reduction capacity of a restriction measure, i.e. its potential to decrease exposure to a level that is considered acceptable and that allows for an adequate control of the remaining risks. Furthermore, the expected costs of the restriction as well as expected risks of possible alternative substances or technologies need be assessed. Moreover, applicants need to document the proposed timeline for reducing exposure to an acceptable level. A restriction is considered ‘proportional’ if it targets the intended reduction of risks in an efficient and cost-effective way, and if restriction effects are well-balanced with the effort and time required by actors for implementing and enforcing it. In addition, it needs to be assessed whether the restriction is compatible with existing legal frameworks (ECHA 2008). Besides being effective, a restriction should be implementable, enforceable and manageable with reasonable effort. Finally, in order to ensure a successful implementation of a proposed restriction, the authority needs to outline how the (intermediate) results of the restriction, e.g. the expected reduction of production levels, the levels of use(s), or expected reduced emission and exposure levels, can be monitored over time.

Although it is not obligatory, most restriction dossiers include an SEA. Following the ECHA guidance document on SEA for restriction processes (ECHA 2008), an SEA can have several, complementary functions as shown in Table 1.

**Table 1:** Scope of SEA in REACH restriction dossiers

Purpose	Explanation
Justification that community-wide action is required	Assessment whether expected positive and negative impacts from the use of a substance are improved/worsened with a community-wide action
Assessment whether the proposed restriction is the most appropriate community-wide action	Comparative assessment of different RMOs regarding their effectiveness, practicality and monitorability
Refinement of the restriction proposal	Assessment whether a modification of the proposed restriction will increase effectiveness, practicality and monitorability of the RMO
Assessment of the proposed restriction	Assessment of benefits and costs under the proposed restriction

*Source* ECHA (2008)

An SEA assesses the expected positive and negative impacts of the proposed restriction, and of one or several alternative RMOs. Such an assessment usually starts with determining a ‘baseline scenario’ (Table S1 in the ESM), which determines the societal risk that is expected to occur in the absence of control measures. The baseline scenario is either a business-as-usual scenario assuming an on-going use of the substance under defined market conditions, or an alternative RMO. The baseline scenario can also include forward looking projections about, for instance, future price levels and demand, expected policy changes or technical innovations facilitating substitution. Restriction dossiers did usually not distinguish between alternative baseline specifications.

The (expected) impacts arising from a particular RMO are assessed against the baseline scenario. Following ECHA (2008), impacts comprise environmental and human health impacts, economic impacts (e.g. net costs of the RMO for producers, manufacturers, importers, downstream users and the society), social impacts (i.e. impacts that do not belong to health and environmental impacts, e.g. change of working conditions) and wider economic impacts (i.e. impacts on trade, competition and economic development). In addition, impacts on the distribution of social costs and benefits within and outside the EU (distributional impacts) should be analysed (Fig. 1).

To conclude about a restriction option’s effectiveness, practicality and monitorability, the different types of impacts need to be quantified and compared against each other. For this purpose, the REACH Guidance on SEA (ECHA 2008) for restrictions suggests different tools (Fig. 1). Cost–benefit analysis (CBA), compliance cost assessment (CCA) and macro-economic modelling require to express impacts in monetary terms. Cost-effectiveness analysis (CEA) and break-even analysis, by contrast, allow some impact categories to be expressed in their natural units, e.g. the number of health incidences observed. CCA captures particularly direct (financial) costs occurring to sectors that are affected by a restriction, which can include cost components belonging to different impact categories (e.g. economic impacts, wider economic and distributional impacts, ECHA 2008). Results of a CCA can also feed in a CBA or in a CEA. A multi-criteria analysis (MCA) is used when robust monetary values do not exist for all impact categories and, thus, quantitative and qualitative impact information have to be considered, weighed and aggregated. Macro-economic models analyse how impacts of a restriction will affect the economy as a whole, for example, through a change of price and income levels or employment. Since they require a large amount of input information, macro-economic models have been considered to be of minor relevance for SEA in REACH restriction procedures (ECHA 2008).

### Plausibility criteria for a comparative evaluation of SEA in REACH restriction dossiers

For the comparative evaluation of SEA in REACH restriction dossiers, we define a set of plausibility criteria, which characterise the conceptual setup, the methodological approach, and the outcomes of SEA. The criteria are listed in Table 2.

**Table 2:** Plausibility criteria for the evaluation of SEA in 24 REACH restriction dossiers

Criteria	Explanation, example(s)
*Conceptual setup of SEA*	
Baseline scenario	Business-as-usual situation
Scope of an SEA	Purpose for which SEA was performed (Table 1)
RMOs considered	Proposed restriction and alternative RMOs
*Methodological approach to impact assessment in SEA*	
Impact categories addressed	Human health impacts, environmental impacts, economic impacts, social impacts, wider economic impacts, distributional impacts (Fig. 1)
Type of IA	E.g. qualitative/quantitative assessment
Method used for impact aggregation/comparison	E.g. cost-effectiveness analysis, cost–benefit analysis, compliance cost assessment (Fig. 1)
Discounting of monetised impacts	Type of discounting method used
Uncertainty analysis	Types of uncertainties addressed and method used for uncertainty analysis (qualitative, deterministic, probabilistic)
*SEA outcomes*	
Main results of a quantitative IA	Results of quantitative impact assessment
Adopted restriction decisions	Comparison of initially proposed, selected and adopted restriction

*IA* impact assessment

### Evaluation of quantitative SEA results

An SEA evaluates different types of impacts arising from the restriction options suggested, and facilitates the selection of the RMO that is ultimately preferred by the dossier submitter. Key quantitative outcomes of an SEA are estimates of a restriction’s expected costs and benefits. Costs of a restriction comprise different components, e.g. compliance costs, substitution costs, testing costs, or costs for training and implementation. Benefits usually consist of avoided health incidents or health damage costs, but can also include increased workplace safety, and incentives for technology development and adoption. In addition, the expected risk reduction capacity of a restriction option, expressed as, for example, the expected emission reduction or the number of avoided health incidences per period (e.g. per year), can be used as a quantitative, non-monetary estimate of benefits. Since a restriction of a substance can have long-term impacts on different societal actors and the environment, cost and benefit estimates presented in SEA can stretch over several years or even decades (Table S3 in the ESM).

Clearly, the properties and uses of the substances addressed in the 24 restriction dossiers differ. Consequently, estimates of the risk reduction capacity, costs and benefits under different restriction options vary widely across dossiers. Using R version 3.4.3 (2017-11-30), we compared the estimates provided for risk reduction capacity, costs and benefits between substances. For our comparison, we distinguished substances classified as SVHC from non-SVHC by a Student’s *t* test. Furthermore, we fitted a linear regression into the data of expected costs and benefits, using the following model:

1 $$\log 10\left( y \right) = a + b*\log 10\left( x \right) + \varepsilon ,$$

where *y* denotes the benefit estimates, *x* the cost estimates, *a* is the intercept, *b* the slope in the linear regression and *ε* is the error term. Statistically significant influence was denoted at a *p* value ≤ 0.05. Prior to the statistical analysis (*t* tests and regression analysis), the variables denoting costs and benefits were log transformed and the arcsin(sqrt(*x*)) from the risk reduction capacity values was calculated in order to achieve a normal/t-distribution.

## Results: Practical implementation of SEA in REACH restriction dossiers

### Conceptual setup of SEA

An overview of the methodological approaches for assessing impacts in SEA, and of the main outcomes of SEA for RMOs that were selected by the dossier submitter, is presented in Table 3. Information on SEA was retrieved from final background documents available at ECHA (2019d). Table S1 in the ESM offers further information about the baseline scenarios, the restriction options identified by the dossier submitter, and, where available, the regulatory decision adopted. Restriction options that the dossier submitter considered inappropriate from the outset (e.g. risk management measures under other European legislations) were not included in the analysis. Depending on the use of the chemical, the societal risk under the baseline scenario was expressed in terms of the amount of consumer articles placed on the market, the number of humans or animals affected, the future production, export or import volume of a substance, the use volume of a substance in consumer articles, or current and future emissions.

**Table 3:** Methods used for impact assessment, number of risk management options (RMOs) addressed in SEA, mean risk reduction capacity, expected costs and benefits of RMOs evaluated in SEA of 22 REACH restriction dossiers

Substance (abbreviation)	Used in branch/product	Method(s) used for assessing impacts	RMO addressed in SEA^a^	Mean risk reduction capacity (%)** (parameter)	Mean expected costs (M€/year), (lower/upper bounds**)	Mean expected benefits (M€/year), (lower/upper bounds^**^); effectiveness estimate	Cost–benefit/cost–effectiveness ratio	Time period of SEA (years)
Lead in jewellery articles (Pb in jewellery)	Jewellery coatings	Break-even analysis of mouthing duration of a piece of Pb, for children between 6 months and 3 years until benefits of avoiding Pb in jewellery articles just exceed costs of the restriction (i.e. expected substitution and testing costs), and human health impacts (lost lifetime earnings due to IQ loss, assuming a mean value of 10,000 € reduction of lifetime earnings per IQ point lost)	1	n.a.^b^	4.6 (2.1–6.3)	Break-even: 1–87 s/child and year (mean 32 s/child and year)	n.a.	1
Phenylmercury compounds (Phenyl-Hg)	Polyurethane coatings, adhesives, sealants and elastomers	Cost-effectiveness analysis, relating the net present value (NPV) in 2010 of compliance costs and the loss of revenue from exports for the entire phase-out period to total expected emissions avoided	1	69 (avoided emissions)	9.7	Total emissions avoided: 14,900 kg	Cost-effectiveness ratio 649 €/kg avoided emissions	19 (2010–2028)
			2	100 (avoided emissions)	14.6	Total emissions avoided: 18,2 kg	Cost-effectiveness ratio 802 €/kg emissions avoided	
Mercury in measuring devices (Hg)	Thermometers (incl. hygrometers), sphygmomanometers (i.e. blood pressure meters), barometers, manometers (incl. tensiometers), metering devices for the determination of softening point, pycnometers, strain gauges used with plethysmographs	Cost-effectiveness analysis for the different measuring devices, relating expected cumulative compliance costs for avoiding Hg to the expected cumulative emission reduction (2015–2034)	1	n.a.^c^	12.3^d^	Emissions avoided: 60.3 t/20 y	Cost-effectiveness ratio 4100 €/kg emissions avoided	20
Phthalates (2012)	Softener of PVC and other plastics, in dispersions, paints and varnishes, carpets, tablecloths, curtains, bags, brief-/suitcases, electric and electronic equipment (EEE), water beds, air mattresses, wallpaper, tapestry, footwear, bathing equipment, balls, etc.	Cost-effectiveness analysis relating total expected substitution costs of raw materials in 2015 to total expected avoided lifetime emissions	1	64 (tonnage reduction)	12.5 (5–20)	Avoided lifetime emissions: 252 t	Cost-effectiveness ratio for different groups of articles: 34.4–366.4 €/kg avoided emissions.	n.a.
Chromium VI compounds (CrVI)	Tanning processes	Partial cost–benefit analysis, assessing compliance costs (tanning and testing costs) and import costs, and health impacts (saved costs due to the prevention of new contact allergy cases, and saved costs for treatment of existing allergy cases).	1	80 (health incidence reduction)	92.5 (85–100)	1.8 (1.5 in year 1)—2.0 in year 20)	n.a.	20
*p*-dichlorobenzene (DCB)	Lab chemical, carrier for textile dyes, crop protection and paper industry, pharmaceuticals, agrochemicals, leather and fabrics, cosmetics, toilet blocks, air fresheners, embalming powder, chemicals of grinding wheels, monomer for the production of polyphenylene sulphide	Partial cost–benefit analysis, assessing expected economic impacts (substitution costs, change of consumer surplus), and avoided health impacts (annualised VoLYL^f^ assuming values of 50,000 € and 120,000 €/year, respectively, and assuming a 20-year assessment period).	1	51 (reduction of exposed population)	2.8 (2.6–3.1)^e^	16.4 (9.6–23.1)	n.a.	20
			2	72 (reduction of exposed population)	3.5 (2.1–5.3)^e^	2.2 (1.3–3.1)		
			3	100 (reduction of exposed population)	1.0 (1.0–2.2)^e^	18.6 (10.9–26.2)		
Lead and its compounds in consumer articles (Pb cons. art.)	Metal alloys, pigments/dyes, as pure metal and as stabiliser in plastic	Partial cost–benefit analysis, assessing expected annualised compliance costs (substitution, testing, and redesign & recycling costs) and expected lifetime benefits of children from reduced Pb exposure (avoided lifetime productivity loss per IQ point decrement, assuming a mean value of lifetime earnings per IQ point lost of 8000 €); break-even analysis of mouthing time.	1	69 (exposure reduction)	34 (12 – 43)	176 (52 – 552)	n.a.	Lifetime^g^
			2	69 (exposure reduction)	91 (24–235)	n.a.		
			3	51 (exposure reduction)	17 (5–20)	n.a.		
			4	n.a.	>40 (24–235)	n.a.		
Nonylphenol varieties incl. ethoxylated nonylphenol (NP/NPE)	Textile and leather auxiliaries, additives in concrete, plastics, food packaging, photographic chemicals, lab chemicals	Assessment of compliance (substitution) costs and benefits (improvement of ecosystem services expressed as reduction of the number of EU countries with risk characterisation ratio of receiving waters > 1)	1	21 (exposure reduction)	45.9	n.a.	6–7 countries in 2021 (compared to 11–12 countries 2010).	11 (2010–2021)
*N*-methylpyrrolidone (NMP)	Solvent and cleaning agent in petrochemical, agricultural, pharmaceutical, electronics and textile industries	Cost assessment (compliance costs, relocation costs turnover affected, lost jobs), health benefit assessment and assessment of reduced number of workers at risk (both confidential and not documented)	1	n.a.	2.5 (1.7–3.3)	n.a.	n.a.	15
			2	86 (exposure reduction)	3.0 (2.7–3.3)	n.a.		
			3	72 (exposure reduction)	19.0 (2.7–35.3)	n.a.		
			4	72 (exposure reduction)	n.a.	n.a.		
Cadmium in artists’ paints (Cd in artists’ paints)	Oil, acrylic, water colours, gouache, pure pigments	Partial cost–benefit analysis, considering consumer costs (financial costs and loss of consumer surplus, administrative costs and costs for discarded products over 50, 100 and 150 years) and health benefits (saved direct, indirect and intangible costs of (i) avoided male and female fractures and (ii) breast cancers, saved monetised QALYs of avoided cancers, assuming loss of 1,65 QALYs per cancer case and a value of 51,000 € per QALY)	1	0.006 (reduced per capita intake CD in food)	0.3 (0.2–0.5)	1.3 (0.6–2.0)	n.a.	150
Chrysotile (CHRY)	Cells for electrolysis, production of chlorine	Assessment of annual costs for baseline A scenario (demand/use in 2015–2025 if the substitute is viable) and a baseline B scenario (substitute is not viable).	1	n.a.	5.8	n.a.	n.a.	10 (2015–2025)
			2	n.a.	0	n.a.		
Bisphenol-A (BPA)	Dye developer in point-of-sales tickets, receipts, self-adhesive labels, lottery tickets, fax paper	Partial cost–benefit analysis, assessing costs (average substitution and compliance costs for different substitution options), and health benefits (NPV of avoided treatment costs for adverse health effects in workers and consumers)	1	70 (avoided env. release)	22.3 (19.3–25.3)	4.3 (3.5–5.2)	n.a.	Health impacts: Lifetime with 2019 as reference year; Costs: 2019-2030
Inorganic ammonium salts (NH_4_)	Cellulose insulation materials	Partial cost–benefit analysis, assessing costs (European technical approvals, other technical approvals, testing) and benefits (avoided costs of re-insulation and re-housing, avoided cost-of-illness based on direct treatment costs in France)	1	95 (health incidence reduction)	0.3 (0.2–0.4)	0.35 (0.3–0.4)	n.a.	24 (2017–2041)
Decabromodiphenyl ether (DecaBDE)	Additive flame retardant in furniture, textiles, in transport, construction and mining sector	Cost-effectiveness analysis relating average substitution costs per year to average expected emission reduction per year	1	100 (emission reduction)	2.2	Average emission reduction: 4.7 t/year	Cost-effectiveness ratio: 464 €/kg avoided emissions	Different timescales for emissions. Production: 2014; Article service life: 10 years; Waste landfills: 30 years
Perfluorooctanoic acid, its salts and PFOA-related substances (PFOA)	Pans and other cooking utilities, photographic applications, semiconductor industry, textiles and leather, firefighting foams, in paper, paints, inks	Cost-effectiveness analysis, relating expected substitution costs after 2015 (operating and investment costs) to expected avoided emissions per year	1	45 (production volume reduction)	17 (1.4 – 121)	Emission reduction PFOA: 5.5 t/year; emission reduction PFOA-related substances: 18.8–55.2 t/year	Cost-effectiveness ratio PFOA: 0–6550 €/kg avoided emissions. Cost-effectiveness ratio PFOA-related substances: 4–3533 €/kg avoided emissions.	Distinction between a ‘current’ and ‘post 2015’ period
Methanol (MeOH)	Paints, varnishes, windshield washer fluids, antifreezes, adhesives, de-icers and cleaning agents	Partial cost–benefit analysis, assessing expected substitution costs and expected benefits (value of avoided fatalities for the period 2004–2011 assuming a VSL of 1,000,000 €), based on data from Finland	1	63 (poisoning incidence reduction)	2.2 (0.4–4.0)	45 (16.1–101)	n.a.^2^	Health impacts: Life year; Costs: 2004–2011
Octamethylcyclotetrasiloxane and decamethylcyclopentasiloxane (D4/D5)	Shampoos, shower-gels, make-up removing products	Partial cost–benefit analysis, assessing annual compliance costs (raw material substitution costs, reformulation costs, product performance reduction costs) and benefits (WTP^h^ for reducing risks to the aquatic environment, assuming a WTP/person of 46 € for D4 and of 40 € for D5); cost-effectiveness analysis, relating annualised compliance costs to the annual expected emission reduction	1	87.5 (emission reduction)	42 (23–61)	19,300; annual emission reduction 199.6 kg/year	115.7–533.4 €/kg	Distinction between a 2- and a 5-year transitional period
			2	n.a.	25 (7.6–42)	19,300; annual emission reduction 199.6 kg/year	28.1–434.5 €/kg	
Silanetriol and any of its mono-, di- or tri-O-(alkyl) derivatives (TDFA)	Water proof articles (textiles, leather, tiles, ceramic), provides water and oil repellence to surfaces such as stone, glass and enamels	Partial cost–benefit analysis, assessing annual compliance (reformulation) costs and benefits (saved direct and indirect costs due to avoided health incidences, assuming a mean cost value of 1870 €/day).	1	n.a.	0.01 (0.008–0.012)	0.3 (0.2–0.5)		n.a.
Phthalates (2017)	Articles including mainly flooring material, coated fabrics and paper, recreational gear & equipment, mattresses, footwear, office supplies and equipment, other articles moulded from or coated with plastic	Cost-effectiveness analysis, relating annual compliance costs (substitution costs, testing costs, recycling costs) and enforcement costs to total amount of phthalates substituted; assessment of expected annual benefits in terms of avoided health damage costs.	1	n.a.	18 (16.9–19.1)	33 (9.8–42.6); amount of phthalates substituted 131,560 t/year	Cost-effectiveness ratio: 130 €/t of substituted phthalate use	20 (2020–2039)
Diisocyanate	Polyurethanes (PU), PU foams assembly foams (e.g. insulation panels), foundry cores (casting), coating materials (paints, lacquers, varnishes), adhesives and glues, elastomers, sealants, pre-polymers in chemical synthesis, engineering plastics, PU fibres	Partial cost–benefit analysis, assessing average annual costs (present value of training and testing costs over a 20-year period) and benefits (present value of avoided direct, indirect and intangible costs from occupational asthma)	1	60 (incidence rate reduction)	95 (26 – 165)	324 (271 – 378)	Benefit–cost ratio 1.65–15 M€/year	20
			2	n.a.	148 (79–218)	324 (271–378)	Benefit–cost ratio 1.2–4.8 M€/year	
			3	n.a.	18126	627	Benefit–cost ratio > 28 M€/year	
Lead-polymers or copolymers of vinyl chloride (Pb-PVC)	Window profiles, cable insulation, pipes and flooring in order to allow the PVC to endure longer fabrication (heating) time and protect it against photo-degradation, thereby prolonging the service life	Cost-effectiveness, relating expected compliance costs (substitution costs) and enforcement costs in 2016 to expected emission reduction in 2016; break-even analysis (number of IQ points to be prevented per year, min. amount of Pb ingested by population per year)	1	60 (reduction PB concentr.)	2.1 (0.9–3.3)	Emissions avoided: 0.35–33.8 t/year	Cost-effectiveness ratio 2484–2499 €/kg (central estimate 308 €/kg); break-even: 209 IQ points/year; 1.24 g/year ingested	Implementation year (2016)
Lead in shot (Pb shot)	Gunshot and other ammunition across a range of sporting, military and law enforcement uses	Cost-effectiveness analysis, relating average annual substitution costs (present value of shotgun replacement costs, operational costs over a 20-year period) to avoided Pb emissions per year; partial cost–benefit analysis, assessing annualised social costs (costs for replacement and testing of guns, new cartridges, reduced by potential producer surplus gains and tax revenues) and annualised benefits (aggregate use value of avoided mortality of waterfowl species)	1	20 (decrease of lead shot ingestion by different waterfowl species)	167 (92–347)	>105; emissions avoided 1432–7684 t/year	Cost-effectiveness ratio 0.3–25 €/kg avoided emissions	3-year transitional period; replacement period shotguns 50 and 20 years, respectively

The dossiers of Cd in paints did not contain an SEA. In the dossier on DMFu, only an explanatory SEA is provided

*Source* Information retrieved from Annex XV Final Background Documents available at ECHA (2019d)

**Values of the scenarios presented in the restriction dossiers. See also Table S1 in the ESM. If lower/upper bounds are missing, values were not provided by the dossier submitter

^a^Not all RMOs identified by the dossier submitter were addressed in the SEA, see also Table S2 in the electronic supplementary material

^b^n.a.: not available

^c^Risk reduction capacity not available for all individual measuring devices

^d^All measuring devices

^e^Assuming a linear demand, upper and lower values reflect different elasticities of demand

^f^VoLYL: value of life years lost

^g^No time period specified in the background document

^h^WTP: willingness to pay

**Fig. 2:**
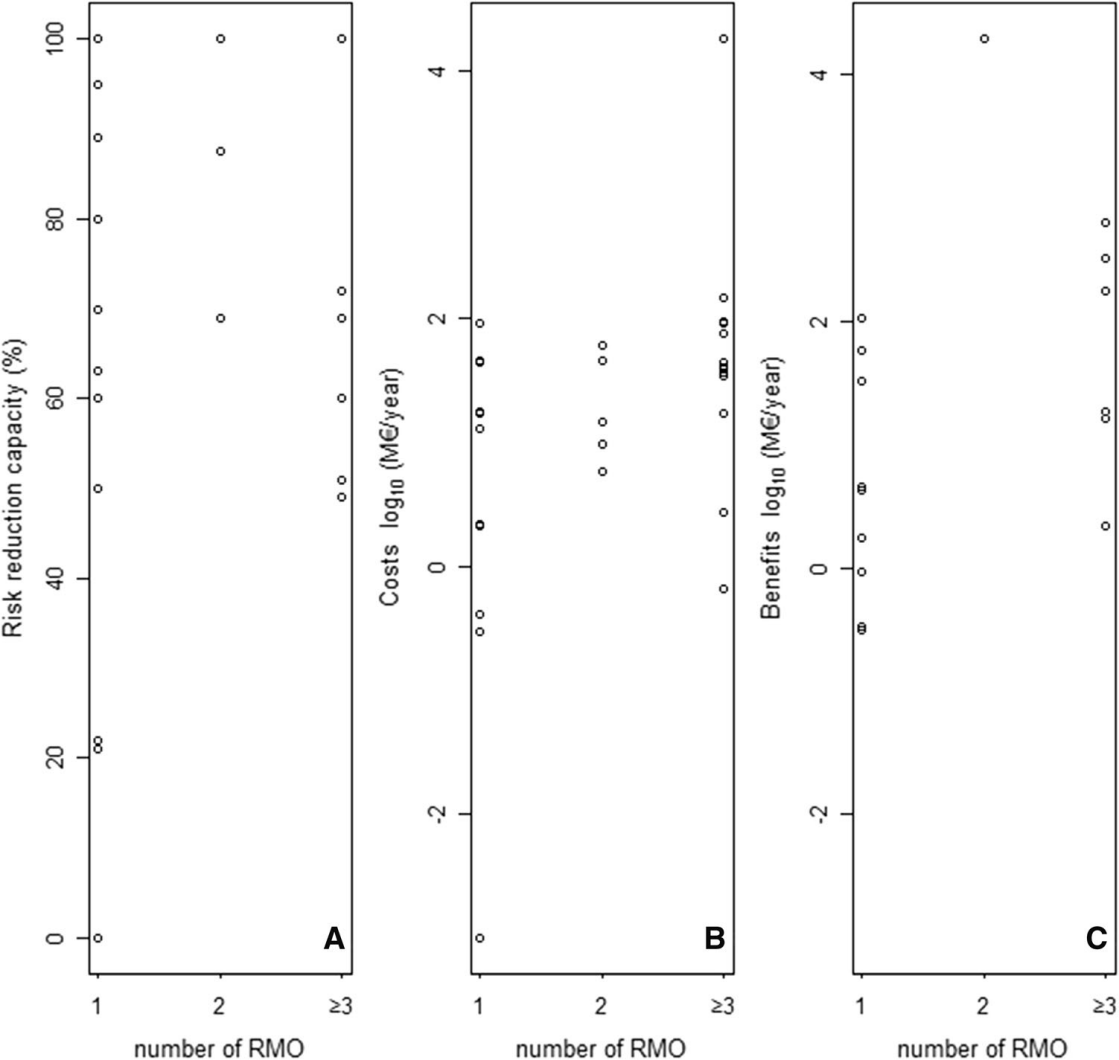
Mean risk reduction capacity (**a**), cost (**b**) and benefit (**c**) estimates of 20 REACH restriction dossiers, grouped according to the number of restriction options (1, 2 and ≥ 3 RMOs) included in the SEA. To make different approaches for assessing restriction options’ risk reduction capacity comparable with each other, the risk reduction capacity was expressed as % improvement compared to the baseline. *Source* Data provided in Table 3

In addition to the initially proposed restriction, REACH restriction dossiers often identified one or more alternative RMOs. However, in several dossiers (e.g. Pb in jewellery, NP/NPE, PFOA, BPA, Pb-PVC, phthalates (2017), NH_4_, Cd in artists' paints), only the preferred (and thus pre-selected) RMO was further evaluated with an SEA (purpose 4 in Table 2, see also Table S1 in the ESM).^1^ Two restriction dossiers (e.g. DMFu, MeOH) proposed only one restriction measure, which then by default became the preferred option.^2^ Restriction dossiers for Phenyl-Hg, DCB, NMP, Pb in consumer articles, diisocyanate, chrysotile, D4/D5 and decaBDE provided a comparative evaluation of the proposed restriction for selected alternative RMOs (Table 3, column 4), which is more comprehensive and, thus, more time and resource consuming than conducting an SEA for individual restriction options only. From the perspective of a dossier submitter, a comparative evaluation seems reasonable if expected social gains and losses are high. Though our analysis did not reveal a clear relationship between the number of restriction options assessed and the quantitative outcomes of SEA (Fig. 2), we find that dossiers providing a comparative evaluation of two, three, or more RMOs document large differences between the different restriction options for at least one outcome parameter (Table 3, columns 5–7).

The risk reduction capacity of an RMO was expressed using estimates of the expected emission reduction, avoided health/poisoning incidences, human or environmental exposure, tonnage reduction, or the reduced concentration of a substance (Table 3, column 5). Due to uncertain or lacking data, impacts were often assessed for different scenarios assuming, for instance, different time spans, alternative economic growth rates, or different estimates of the number of people affected. For RMOs with a quantitative impact assessment we present the mean values, and the lower and upper bounds of cost and benefit estimates (Table 3, columns 6 and 7).

### Methodological approach to impact assessment in SEA

For assessing impacts of an RMO, the dossier submitters used partial CBA, CEA, CCA and break-even analysis. MCA and macro-economic modelling were not applied in SEA. Generally, impacts can be assessed in a qualitative (descriptive) or in a quantitative manner. Furthermore, quantitative impact assessments can either be expressed in monetary or non-monetary terms.

**Fig. 3:**
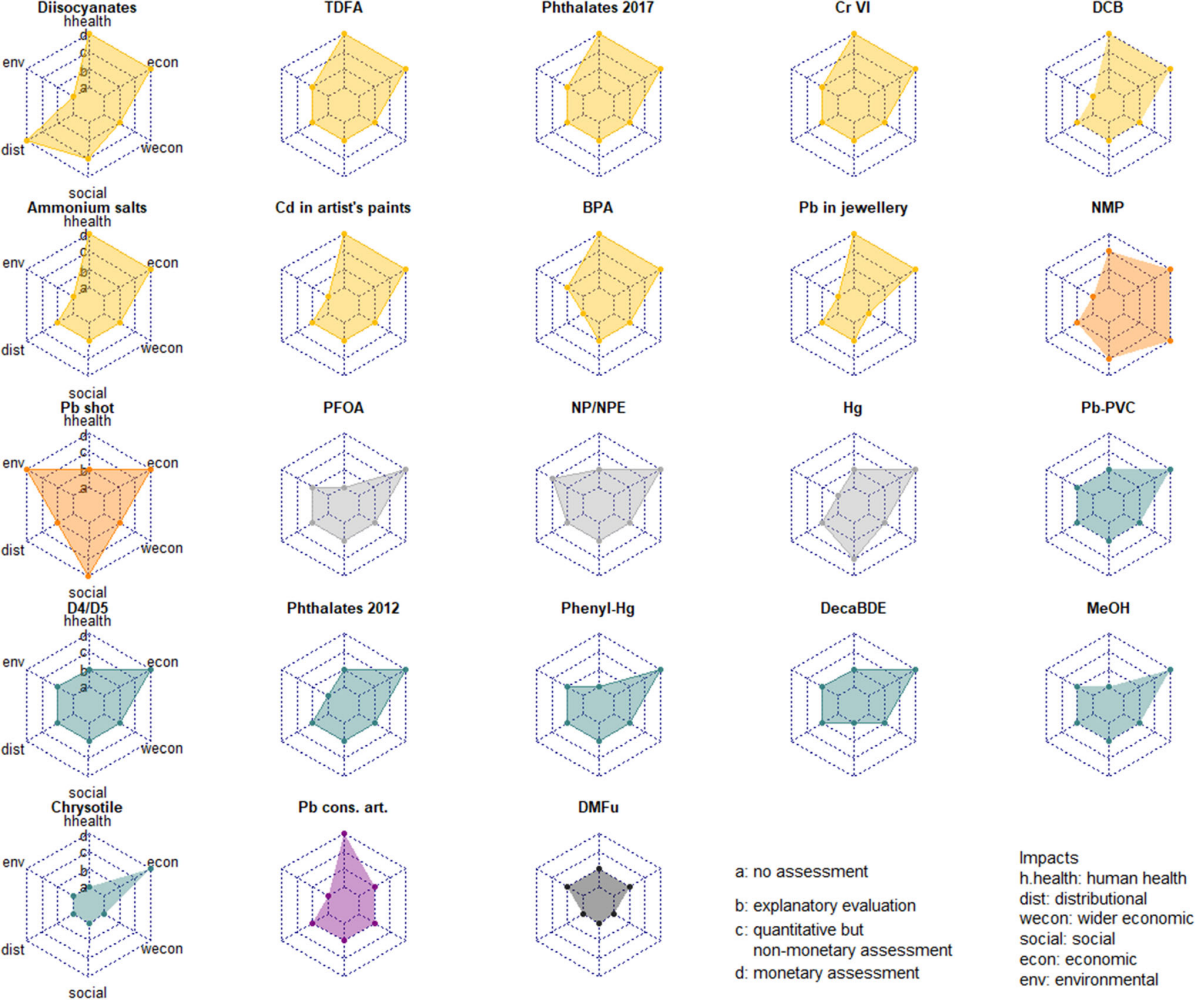
Approaches to impact assessment in SEA of 23 REACH restriction dossiers. Radar charts of restriction dossiers with a similar methodological approach to impact assessment have the same colour. *Source* Annex XV Final Background Documents available at ECHA (2019d)

Figure 3 illustrates the type of impact assessments for 23 of the 24 REACH restriction dossiers considered. The restriction dossier for Cd was excluded because it did not contain an SEA. For each dossier, we indicate whether a certain impact category was assessed or not, and what types of approaches to impact assessment were applied (explanatory, quantitative but non-monetary, monetary assessment).

Overall, we observe that the routines for impact assessment vary considerably across restriction dossiers. In 16 of 23 dossiers, only selected impacts were assessed, i.e. one or more impact categories were not addressed. Economic impacts were quantified and monetised in 21 dossiers. In case of human health impacts, 10 of 23 dossiers included monetary assessments of human health impacts. Specifically, the analyses provided estimates of expected (avoided) health damage costs given the adoption of the selected restriction measure (Table 3, column 3).

Economic impacts were predominantly assessed as financial costs, in particular direct compliance or implementation costs (Table 3, column 3). In addition, dossier submitters presented, where possible and appropriate, information about indirect compliance costs such as price changes due to a change of product quality. Compliance costs were often used as approximation of economic impacts. Only few dossiers (Phenyl-Hg, DCB, Cd in artist paints, phthalates 2017, Pb in gunshots) adopted an approach to assessing economic impacts that includes, besides financial costs, opportunity cost components (monetary estimates of foregone opportunities for resource use (OECD 2002), including enforcement costs and expected changes of consumer or producer surplus).

Compared to economic impacts, quantified health and environmental impacts capture the (avoided) external costs and, thus, the benefits of a restriction option in comparison to the baseline scenario. Two dossiers (Pb in shot, NP/NPE) offered a quantitative assessment of (avoided) environmental impacts, i.e. NP/NPE and Pb in shot (Table 3). In the latter dossier environmental impacts were assessed as avoided opportunity costs of water birds saved by the restriction. The restriction dossier for NP/NPE adopted a simplified ecosystem services approach, using the change of the number of EU member states with a risk characterisation ratio (RCR) in freshwater of < 1 as a proxy for an overall improvement of ecosystem services. A qualitative (explanatory) assessment of environmental impacts was presented in the dossiers TDFA, phthalates 2017, CrIV, BPA, Pb-PVC, D4/D5, Phenyl-Hg, decaBDE, MeOH, and DMFu. Several dossiers (diisocyanates, DCB, NH_4_, Cd in artist paints, NMP, Hg, Pb, CHRY, Pb in jewellery) did not provide an environmental impact assessment because it was considered beyond the scope of the restriction, or due to a lack of information (Fig. 3). Similarly, social, wider economic and distributional impacts were predominantly evaluated qualitatively, and remained unassessed in three dossiers.

For assessing health impacts, different approaches were used. Several dossiers (Cr IV, D4/D5, TDFA, Diisocyanate, Phthalates 2012) quantified health impacts as avoided health damage costs, which were assessed as saved direct costs (cost-of-illness, health treatment costs) and indirect costs (avoided productivity loss, saved losses of lifetime earnings). Where health impacts could not straightforwardly be expressed as (saved) costs, impact parameters related to morbidity and mortality such as the number of fatalities, or utility-based measures such as Quality Adjusted Life years (QALY) or Value of a Life Year (VOLY) estimates and IQ points lost were monetised using available information on, for example, the willingness to pay (WTP) for avoiding a health incidence, or money values per QALY/VOLY (Pb in jewellery, DCB, Cd in artists' paints, Pb in consumer products, MeOH).

Where health and environmental impacts were neither monetised nor quantified, estimates of the expected reduction of emissions were used as proxy of avoided impacts and, thus, the benefits of a restriction option. Note that the change of emissions also served as a proxy for a restriction option’s risk reduction capacity, being a key component for evaluating the effectiveness of a restriction option (Fig. 1).

Obviously, impact assessment in an SEA is forward looking. Expected costs and benefits usually stretch over a time period, and can occur in different periods. To make estimates of benefits and costs of a restriction option comparable in an intertemporal setting they need to be discounted to their present values (Heal 2007). In the economics literature, different discounting approaches have been discussed, of which exponential discounting (using a constant discount rate) and hyperbolic discounting (using a declining discount rate) are most prominent (Samuelson 1937; Osborne 2016). The REACH Guidance for SEA in restrictions (ECHA 2008) offers an overview of discount rates applied at the EU level (e.g. in the Impact Assessment Guidelines of the EC, European Commission 2019) and at the national level of EU Member States. Furthermore, a sensitivity analysis using different discount rates is recommended if the time span of expected costs and benefits to occur exceeds 30 years. Following the recommendations in the impact assessment guidelines of the EC (2019), the majority of restriction dossiers adopted exponential discounting using a standard discount rate of 4% for both cost and benefit estimates (Table S3 in the ESM). Two dossiers (Cd in artists' paints and BPA) offered a critical discussion of the impacts of discounting on costs and benefit assessments. These dossiers addressed restrictions on substances with a clear inter-generational spread of costs and benefits (i.e. > 30 years). For the assessment of avoided health damage costs, a higher discount rate (e.g. 3.5 or 4%) was applied for early years, whereas a lower discount rate (2%) was used for late years of the assumed time period. Eight dossiers (Phthalates 2012, Cd in paints, DFMu, decaBDE, PFOA, MeOH, D4/D5, Pb shot) did not apply or discuss discounting, although they addressed substances with a clear long-term impact profile. Though there are arguments for using (very) low positive or even negative discount rates if impacts are expected to occur far in the future (Weitzman 1998; Fleurbaey and Zuber 2013), the lack of discounting was often not motivated in the dossiers (decaBDE, PFOA, D4/D5).

Given the complex hazard and use profiles of substances addressed in REACH restriction processes, impact estimates provided in restriction dossiers are subject to different types of uncertainties. Our analysis revealed that parameter uncertainty (referring to the value of a parameter used in SEA, e.g. emissions, production/import/export volumes, cost parameters, the number of people exposed, the price elasticity of demand, the implementation period), model uncertainty (referring to the setup of an assessment, e.g. the shape of the market demand curve) and legal uncertainty (e.g. implementation of authorisations) were most frequently discussed (Table S3 in the ESM). The REACH Guidance document (ECHA 2008) suggests addressing uncertainties by applying sensitivity analysis, scenario analysis, or expert judgement. Of the 24 dossiers analysed, 20 applied a quantitative deterministic uncertainty analysis, being either sensitivity analysis or scenario analysis. None of the dossiers applied probabilistic methods (e.g. Monte Carlo Analysis) for uncertainty analysis.

### SEA outcomes

In most restriction dossiers, the preferred control measure was equivalent to the proposed restriction option (Table S1 in the ESM). Exemptions are the dossiers on chrysotile and PFOA, its salts and PFOA-related substances. For chrysotile, a restriction had already been in place before REACH entered into force, however, Member States had granted exemptions until 2025. In the restriction dossier, the submitter suggested a continued use of chrysotile as preferred RMO, based on a binding agreement with users to fully substitute chrysotile until 2025. In case of PFOA, the dossier submitter preferred a full ban to a ban with possible derogations (ECHA 2019d, Table S1 in the ESM). Furthermore, dossier submitters did not always base the selection of the preferred RMO on the outcomes of SEA. For instance, although RMO 2 showed a superior cost-effectiveness ratio than RMO 1 in the D4/D5 dossier (Table 3, column 8), the latter was selected as it was considered proportionate with the cost-effectiveness ratios estimated in other dossiers (Table S1 in the ESM, ECHA 2019d).

As illustrated in Fig. 3, not all impact categories addressed in an SEA were assessed quantitatively. Where economic, and health and/or environmental impacts were quantified and monetised, dossier submitters conducted a partial CBA (Pb cons, Cd in artist paints, DCB, BPA, Cr IV, MeOH, D4/D5, Diisocyanate, TDFA, Pb in shot, Table 3). Cost and benefit values were not always combined to a net present value (NPV) estimate, but documented separately. Where health and/or environmental impacts could be quantified but not monetised, CEA was applied, relating the expected costs of a restriction option to expected annual or lifetime emissions avoided (Phenyl-Hg, Hg in measuring devices, phthalates (2012 and 2017), decaBDE, PFOA, Pb-PVC, D4/D5, Pb in shot). Two dossiers (Pb in jewellery and Pb-PVC) also offered a break-even analysis for assessing health impacts. In some cases (NP/NPE, NMP, chrysotile), the dossier submitter considered health or environmental impacts to be beyond the scope of the proposed restriction or to be of only marginal relevance and, thus, gave focus to economic impacts using a CCA.

**Fig. 4:**
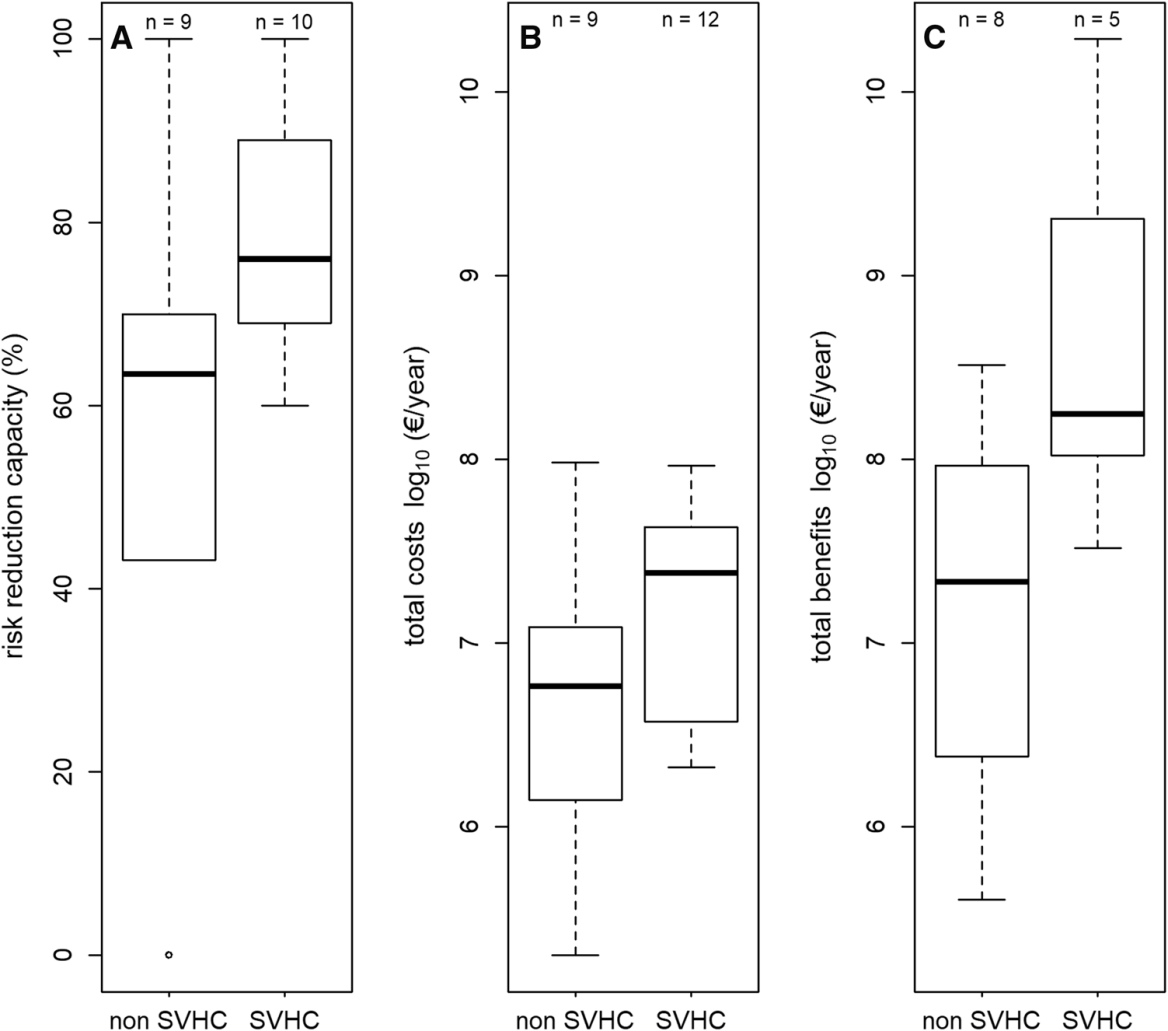
Risk reduction capacity (**A**), costs (**B**) and benefits (**C**) of substances classified as Substances of Very High Concern (SVHC) and non-SVHC. Sample sizes differ between panels due to lacking quantitative estimates for the different parameters. In each panel the subgroup size is indicated above every boxplot. The upper and lower ends of the box represent the 25th and 75th percentiles, the horizontal line in the box the median, and the upper and lower whiskers are the 10th and 90th percentiles, respectively. The dot in panel A is an outlier. *Source* ECHA (2019d), own calculations

Figure 4 compares key quantitative outcomes of an SEA, i.e. the restriction option’s risk reduction capacity (panel A), costs (panel B) and benefits (panel C) between substances classified as SVHC and non-SVHC. We find that estimates of the risk reduction capacity, the expected costs and the expected benefits of an RMO are usually higher for dossiers addressing SVHC. For the expected benefits of a proposed RMO this effect is significant (*p* value 0.032).

This may be explained by considering that SVHC are substances which, according to their properties, are likely to cause long-term impacts to the environment or to human health. These impacts can easily outweigh the costs of a restriction that occur in a limited time period. In addition, the uncertainty regarding the size and the occurrence of impacts is often high (Daston et al. 2003; Brouwer et al. 2016). This may cause benefit assessments, being the social gains of a restriction and expressed in terms of the risk reduction capacity, or as avoided health and environmental impacts, to be overstated in comparison to cost estimates, denoting predominantly the financial costs of firms.

**Fig. 5:**
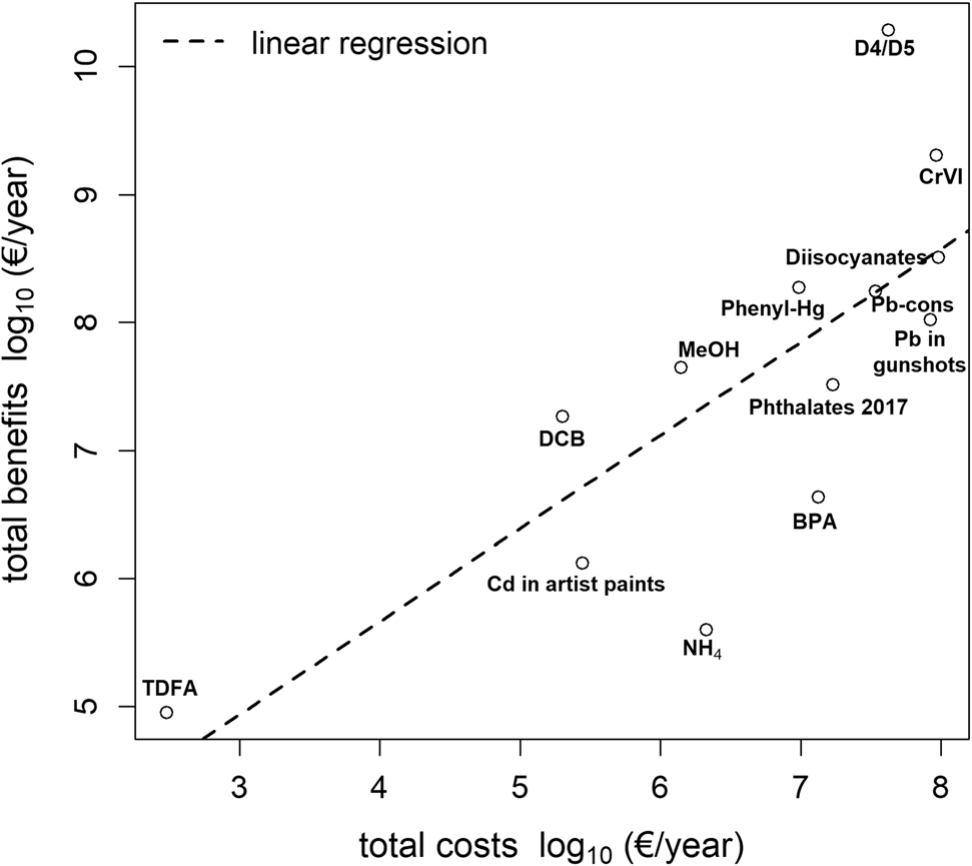
Benefits versus cost estimates retrieved from 13 REACH restriction dossiers. *Source* ECHA (2019d), own calculations

Finally, using linear regression, the benefits of a restriction were related to costs reported in 13 restriction dossiers (Fig. 5). We find a significant and positive linear relationship between expected log_10_ costs and log_10_ benefits (see Table S2 in the ESM, *p* value of 0.0026). This suggests that dossier submitters are willing to accept higher costs for restrictions if substances that are expected to reveal relatively higher benefits.

For the majority of dossiers, the EC adopted the restriction that was identified as preferred option by the dossier submitter, however, following the comments of the RAC and/or SEAC, modifications were added in several cases (Table S1 in the ESM). In few cases (Pb in jewellery, NMP, PFOA), the decision adopted by the EC deviated from the RMO preferred by the dossier submitted.

The restriction dossiers of two substances, phthalates 2012 and Cd in artists' paints, were rejected by the EC because the necessary conditions provided by REACH Art. 68 were considered not to be fulfilled. For TDFA, Diisocyanates, Pb-PVC and Pb in shot opinions from RAC and SEAC have been adopted, however, final decisions of the EC are still pending (Table S1 in the ESM, ECHA 2019d).

## Discussion

This paper is, to the best of our knowledge, the first offering a systematic and critical review of the methodological setup of SEA and its practical implementation in REACH restriction dossiers.

Considering the overall goal of REACH restrictions, i.e. establishing effective risk management measures for industrial chemicals at a Community-wide level, an SEA should adopt a social welfare perspective. This requires impact assessment in SEAs to be comprehensive and comprehensible. Our analysis shows, however, that the current scope of SEA in restriction dossiers has been rather narrow. Specifically, quantitative assessments were performed predominantly for selected impact categories, in particular economic and health impacts, and evaluated a restriction option’s effectiveness. Furthermore, the assessment of economic impacts focused on direct (financial) costs such as compliance and substitution costs. Similarly, the few dossiers quantifying avoided direct and indirect health damage costs relied on existing estimates of medical treatment costs, morbidity and mortality cost estimates available in the scientific and policy literature. Environmental, social, distributional and wider economic impacts were either evaluated qualitatively or remained unassessed.

There may be several reasons for the fragmentary assessment of impacts. First, existing evidence on the properties and risks of individual substances is often incomplete or inconclusive. Relevant in-house data of companies are usually confidential and not accessible for dossier submitters. This complicates developing a distinct list of, for example, health and environmental impacts, and prioritising impacts that are most relevant for the scope of a restriction dossier. Second, for several impact categories (health, environmental, distributional impacts), there is a lack of monetary values that allow transforming impacts into benefit and cost estimates. Generating these values, for example by performing a restriction-tailored willingness-to-pay or remediation cost study, is time- and resource-consuming and therefore not feasible within the time frame of a restriction procedure, and the personal endowment of regulatory agencies. Fourth, our review points to a fundamental lack of ready-for-use approaches for a concern-based impact assessment. For instance, while for some pollutants (e.g. metals, see Giang and Selin 2016; Nedellec and Rabl 2016; pesticides, see Fantke et al. 2012) health impact assessment approaches have been developed and applied, for most chemicals and endpoints the necessary methodological groundwork and the analytic routines for assessing environmental and health impacts are lacking (Gabbert 2017; Holland 2017a, b; Hunt and Dale 2018). Consequently, health and environmental impact assessment in REACH restriction dossiers largely remained at the level of standard environmental risk metrics, e.g. the expected change of the risk characterisation ratio, or estimates of avoided emissions as proxy of avoided environmental and health impacts. While emissions are an obvious starting point for assessing exposure and, thus, environmental and health impacts, they do not always adequately capture the regulatory concerns related to the chemicals addressed in REACH restriction processes. For example, several dossiers addressed chemicals which meet the criteria for being classified as PBT/vPvB (e.g. PFOA, D4/D5, decaBDE). As argued by Gabbert and Hilber (2016), these chemicals are stock pollutants, i.e. they can accumulate in the environment and, therefore, an unregulated release can cause long-term impacts to ecosystems and to human health. Hence, the relevant variable for assessing impacts is the stock—i.e. expected environmental concentrations—during the release period *and* the period after regulatory measures have been adopted (see Gabbert et al. (2017) for a detailed discussion). Since the stock can remain in the environment even long after emissions have ceased, estimates of avoided annual emissions ignore long-term impacts and will likely overestimate expected benefits of a restriction. Consequently, cost-effectiveness ratios of restriction options based on emission estimates, and comparisons of such ratios across restriction options or dossiers are of little informational value and can even be misleading.

While most dossiers identified two or more restriction options, only few dossiers performed a comparative SEA. Interestingly, the preferred restriction option was not necessarily the one with the most favourable estimate of benefit–cost ratio, or with the lowest cost per unit of effect (e.g. per kg emission reduction). This implies that the selection of the preferred option was largely independent of the results of SEA.

## Conclusions

Of the 24 REACH restriction dossiers considered in this paper, 23 included an SEA. This underlines that SEA, though according to Article 68 of the REACH legislation (EC 2006) not being mandatory, is considered important for the evaluation of REACH restriction options, in particular of their effectiveness. However, despite the remarkable effort that dossier submitters have put in conducting an SEA, its current contribution in REACH restriction dossiers boils down to synthesising existing knowledge on selected impacts, benefits and costs.

Considering that, complementary to REACH authorisations, restriction procedures are a key regulatory instrument towards a safe use of chemicals in Europe and beyond (Coria 2018; Georgiou et al. 2018), the function of SEA as a decision-support tool could and should be strengthened. First, attention should be given to improving the quality of impact assessments in an SEA. In particular, there is an urgent need to developing integrated methods which allow for a concern-based evaluation of impacts. Such methods must link environmental assessments (toxicological hazard and risk assessment, environmental fate and distribution modelling, exposure assessments, environmental monitoring) with economic approaches addressing the assessment and valuation of impacts, and with tools that guide the aggregation and comparison of impacts. This requires substantial investments into research and capacity building.

Second, the selection of the preferred restriction option should emerge from its relative superiority in relation to alternative RMOs. Thus, a comparative SEA, even if not being required by law, should become the default in order to improve the transparency and credibility of the restriction decision, and to increase the value of SEAs for regulatory decision-making.

## Electronic supplementary material

Below is the link to the electronic supplementary material.

Supplementary material 1 (PDF 360 kb)

## Footnotes

1 See Hilber and Gabbert (2019) for a detailed analysis of the RMO selection process.

2 Recent restriction dossiers (e.g. phthalates (2017), diisocyanates (2017), and Pb-PVC (2017)) have adopted a new format. Specifically, the organisation of the reports changed from a topic-related approach (with separate sections for the presentation of the proposed restriction (A), hazard and exposure assessment (B), alternative substances information (C), the justification for action on a Union-wide basis (D) and the justification why the proposed restriction is the most appropriate (E), SEA of the proposed restriction (F)) to a process-oriented approach where the evaluation of RMOs including SEA has been consolidated into one section “Impact assessment”.
