# Supplementary material for: Socio-economic analysis in REACH restriction dossiers for chemicals management: A critical review
Source: Ambio. 2019 Dec 5;49(8):1394–411. doi: 10.1007/s13280-019-01285-9 (PMC7239982; doi:10.1007/s13280-019-01285-9)
Supplement: Supplementary file 1 — Supplementary material 1 (PDF 360 kb) [file 13280_2019_1285_MOESM1_ESM.pdf]

Socio-economic analysis in REACH restriction dossiers for chemicals management: A critical review

Electronic Supplementary Material

Ambio - forthcoming

Dr. Silke Gabbert (corresponding author), Wageningen University & Research, Department of Social Sciences, Environmental Economics and Natural Resources Group, Hollandseweg 1, 6700 EW Wageningen, The Netherlands.

Phone: +31 317 483870, Email: silke.gabbert@wur.nl

Dr. Isabel Hilber, Agroscope, Reckenholzstrasse 191, 8046 Zurich, Switzerland

Table S1: Basic information of 24 REACH Annex XV restriction dossiers[†] (substance name, CAS number, submitting authority, SVHC classification), baseline scenario and risk management options (RMOs) specified by the dossier submitter, and regulatory decision adopted by the European Commission.

No.	Substance[a] (CAS Nr.)	Submitting authority (date of dossier submission)	SVHC[b]	Baseline scenario	RMOs considered (proposed/preferred restriction option in bold)	Regulatory decision
1	Pb in jewellery (7439-92-1)	French Competent Authority (04/2010)	yes	Placing on the market of about 150 million pieces of jewellery articles, with an average Pb content of 6% and with a growing trend due to growing market for imports of fashion jewellery.	RMO 1: Restriction on the use and placing on the market of fashion jewellery based on the Pb migration rate;<br>RMO 2: Restriction on the use and placing on the market of fashion jewellery based on Pb content;<br>RMO 3: Restriction on the use and placing on the market of fashion jewellery based on the Pb migration rate AND Pb content;<br>RMO 4: Ban on Pb and its compounds in fashion jewellery which are used and placed on the market;<br>RMO 5: Ban on Pb and its compounds in some fashion jewellery which are used and placed on the market;<br>**RMO 6 (proposed and preferred option)**: Restriction on the use and placing on the market of jewellery (fashion and precious) based on the Pb migration rate;<br>RMO 7: Restriction on the use and placing on the market of jewellery (fashion and precious) based on the Pb content and the Pb migration rate.	Adoption of RMO 2, with modification as suggested by RAC/SEAC: Pb concentration being equal or greater than 0.05% by weight.
2	Phenyl-Hg (Phenylhg acetate 62-38-4 phenylhg propionate 103-27-5 phenylhg 2-ethylhexanoate 13302-00-6 Phenylmercuric octanoate 13864-38-5 Phenylmercury neodecanoate 26545-49-3)	Norwegian Climate and Pollution Agency (06/2010)	no	Exponential decrease of Phenyl-Hg use and export compared to the EU use profile in 2008.	**RMO 1 (proposed and preferred option)**: Phase-out of 5 phenyl-Hg compounds in concentrations above 0.01% Hg weight by weight after 5 years;<br>RMO 2: Phase-out period of 5 phenyl-Hg compounds after 2 years.	Adoption of RMO 1.

3	Hg<br>(7439-97-6)	ECHA<br>(06/2010)	no	Total annual amount of Hg in measuring devices for industrial and professional uses placed on the market (as approximation of the maximum emission potential) 13-63.4 t/y.	**RMO 1 (proposed and preferred option)**: Hg containing barometers, hygrometers, manometers, sphygmomanometers, strain gauges, tensiometers, thermometers and other non-electrical thermometric applications shall not be placed on the market after 18 months of restriction entering into force;<br>Alternative RMOs defined for some measuring devices.	Adoption of RMO 1 with derogations depending on the specific device.
4	Phthalates (2012)<br>(Diisobutyl phthalate (DIBP) 84-69-5<br>Dibutyl phthalate (DBP) 84-74-2<br>Benzyl butyl phthalate (BBP) 85-68-7<br>Bis(2-ehtylhexyl)phthalate (DEHP) 117-81-7)	Danish Competent Authority for REACH, Danish Environment Protection Agency<br>(08/2011)	yes	Estimated total amount of phtalates in articles in 2015 (132.000 t) and in 2020 (113.000 t). Assumed substitution rate of 3% for all articles.	**RMO 1 (proposed and preferred option):** Restriction on the placing on the market of all articles intended for indoor use and that contain one or more of the four phthalates DEHP, DBP, BBP and DIBP in concentrations ≥ 0.1% of any plasticised parts;<br>RMO 2: Restriction on the placing on the market of all articles which contain one or more of the four phthalates DEHP, DBB, BBP or DIBP in a concentration ≥ 0.1% (w/w) of any plasticised material. Exemptions will apply for articles solely used outdoors (including storage);<br>RMO 3: Restriction on all articles;<br>RMO 4: Restriction on identified groups of articles;<br>RMO 5: Migration based restriction;<br>RMO 6: Restriction on the placing on the market of consumer articles and any construction material to be used (including storage) in interiors/indoors containing one or more of these phthalates in a concentration ≥ 0.1% by weight of any plasticised material (with certain derogations).	Restriction proposal not considered justified by RAC and SEAC.
5	CrVI<br>(n.a.[d])	Danish Competent Authority for REACH, Danish Environment Protection Agency<br>(01/2012)	yes	Approx. 0.5 million people will suffer from allergic contact dermatitis due to exposure to Cr VI over a 10-year time period. The yearly number of new chromium allergy cases from leather is expected to be 16,875.	**RMO 1 (proposed and preferred option):** Restriction on the placing on the market of articles of leather containing Cr VI in concentrations ≥ than 3 mg/kg of the total dry weight of leather;<br>RMO 2: Restriction of Cr VI in all articles of leather;<br>RMO 3: Restriction of total chromium content of leather.	Adoption of RMO 1 with a compliance period of 12 months.

6	DCB<br>(106-46-7)	ECHA<br>(04/2012)	no	Use of DCB in air fresheners and toilet blocks in the EU is expected to decline from about 4500 t in 1994 to <1000 t in 2012. Expected EU population at risk (exposure > DNEL[c]) is approx. 80,850 in 2012	RMO 1: Restriction on the placing on the market of DCB based air fresheners and toilet blocks for consumer use;<br>RMO 2: Restriction on the placing on the market of DCB based air fresheners and toilet blocks for professional use;<br>**RMO 3 (proposed and preferred option)**: Restriction on the placing on the market of DCB based air fresheners and toilet blocks for both consumer and professional use.	Adoption of RMO 3 with compliance period of 12 months and with modification: Restriction applies to DCB in concentration of min 1% DCB by weight.
7	Pb cons. art.<br>(7439-92-1)	Swedish Chemicals Agency<br>(12/2012)	yes	Estimated annual exposure of children aged 6 – 36 months in the EU is assumed to be 367 g Pb/y, resulting in a total estimated IQ loss of 32,000 points/y.	**RMO 1 (proposed and preferred option)**: Restriction of Pb content in articles and part of articles, that are sold to the general public and that can be mouthed by children;<br>RMO 2: Restriction of Pbd migration in articles and part of articles, that are sold to the general public and that can be mouthed by children;<br>RMO 3: Restriction of Pb content in (all accessible parts of) clothes, accessories and shoes;<br>RMO 4: Restriction of Pb migration in all articles and part of articles that are sold to the general public.	Adoption of RMO 1 with limit content of lead for metallic and non-metallic parts of articles concentration limit ≥ 0.05% (w/w).
8	NP/NPE<br>(n.a.)	Swedish Chemicals Agency<br>(07/2013)	yes	Decrease of emissions to waste water from 2010 to 2015 due to assumed reduction in emissions from other sources than textiles in combination with improved WWTP's[c], but increase of emissions from 2015 to 2031 due to an assumed growth in emissions from imported textiles. Emissions of NP/NPE to surface water are considered to remain relatively stable.	**RMO 1 (proposed and preferred option)**: Restriction of NP/NPE use in textile articles, or textile parts of articles, above a concentration limit of ≥ 0.01% by weight and with a transitional period of 5 years;<br>RMO 2: NPE Limit value < 0.01% by weight for textile articles, or textile parts of articles, with a transitional period of 5 years.	Adoption of RMO 1.
9	NMP<br>(872-50-4)	RIVM<br>The Netherlands<br>(09/2013)	yes	Estimated trends of NMP uses for the period 2011-2016 (not published), compared to NMP production volume in EU in 2005 (20,000-30,000) tons.	RMO 1: Restriction on the manufacture, placing on the market and use of NMP for all applications in concentrations ≥ 0.3% by weight (total ban);<br>RMO 2: Restriction on NMP as substance and in mixtures with a derogation under specific conditions for the use in specific industrial applications;	Adoption of RMO 1 with modification (adjusted DNEL) following the recommendation of RAC.

					**RMO 3 (proposed and preferred option)**: Restriction on manufacture and use if exposure/DNEL is > 5/10/20 mg/m^3<br>RMO 4: Authorisation.	
10	Cd in paints<br>(7440-43-9)	ECHA<br>(10/2013)	no	Projected amounts of Cd in antifouling paints are expected to grow from 256 kg (2012) to 312 kg (2022).	**RMO 1 (proposed and preferred option)**: Modification of the current entry 23: Restriction on the placing of the market of paints containing Cd with concentration limit of ≤ 0.01%;<br>RMO 2: Restriction on the placing of the market of paints containing Cd with concentration limit of ≤ 0.01%, with derogation for copper-based antifouling paint with concentration limit of ≤ 0.0175%.	Adoption of RMO 1.
11	Cd in artists' paints<br>(7440-43-9)	Swedish Chemicals Agency<br>(12/2013)	no	Amount of Cd in artists' paints used in the EU will be 6.4 t/y.<br>Cd concentration in agricultural soils is expected to decrease by 1.6%/y.<br>Per capita intake of Cd via food is estimated to be reduced by 0.0010 µg Cd day^{-1} (baseline 15.2 µg Cd day^{-1}) over a period of 100 years.<br>Number of human bone fractures per year caused by baseline content of Cd in artists' paints is assumed to be 37 for females and 11 for males.	RMO 1: Restriction on the placing on the market and use of Cd based artists' paints;<br>**RMO 2 (proposed and preferred option)**: Restriction on the placing on the market and use on Cd based artists' paints, with an exemption for restoration and maintenance of historical pieces of art.	Commission considered the necessary conditions following Art. 68 of REACH not to be fulfilled. Restriction proposal not adopted.
12	CHRY<br>(12001-29-5<br>132207-32-0)	ECHA<br>(01/2014)	no	Baseline A: Dow will adopt CHRY-free alternative over 2015-2025.<br>Baseline B: Alternative technology does not prove to be technically or economically viable and Dow continues to use CHRY under the existing exemption in entry 6.	**RMO 1 (proposed option)**: Continuation of the current derogation from prohibiting the manufacture, placing on the market and use of chrysotile fibres and of articles and mixtures containing these fibres with time-limited exemptions of national legislations (10 years). Exemption can be renewed;<br>**RMO 2 (preferred option)**: Derogation from prohibition with a fixed end date (2025) as specified in entry 6. Exemptions can principally be extended via a regular Annex XV restriction procedure.<br>RMO 3: Same as option 2, but additional specification of a volume constraint for the exemption.	Adoption of RMO 2.

13	DMFu<br>(624-49-7)	French Competent Authority<br>(04/2014)	no	The temporary ban under the product safety direc-tive (EU Decision 2009/251/EC and prolonged by Commission Decisions 2010/153/EU and 2011/135/EU until March 15th 2012) shall not be renewed and no other EU or national regulation on the use of DMFu shall be introduced.	**RMO 1 (proposed and preferred option)**: DMFu shall not be used in articles in concentration >0.1 mg/kg. Articles containing DMFu >0.1 mg/kg shall not be placed on the market.	Adoption of RMO 1.
14	BPA<br>(80-05-7)	ANSES France<br>(05/2014)	no	Production volume of thermal paper is about 1.2-1.6 million tons in 2005/2006, no imports, export > 0.065 million tons (2005/2006), consumption of thermal paper is about 1.1 million tons (2006 and 2010).<br>Expected trends:<br>• A growing market of thermal paper<br>• An expected ‘spontaneous’ decrease in the use of BPA in thermal paper<br>• An already underway substitution of BPA by alternative developers in thermal paper	**RMO 1 (proposed and preferred option)**: Limitation of BPA in thermal paper to concentrations equal or smaller than 0.02% by weight, by either switching to substitute BPS[f] or to non-bisphenol alternatives. The transitional period proposed for the entry into force is 3 years;<br>RMO 2: Limitation of the migration of BPA in thermal paper into coat thermal paper to avoid absorption by contact.	Adoption of RMO 1.
15	NH_4<br>(ammonium sulphate<br>7783-20-2<br>ammonium dihydrogenorthophosphate 7722-76-1<br>diammonium hydrogenorthophosphate 7783-28-0)	ANSES France<br>(06/2014)	no	Continued use of ammonium salts in insulation, which would result in estimated 300 exposed persons in the EU in 2017	**RMO 1 (proposed and preferred option)**: Restriction of ammonium salts emissions in cellulose insulation materials unless emission of ammonia gas of such materials is < 3 ppm.<br>RMO 2: Restriction on ammonium salts content (composition-based restriction);<br>RMO 3: Authorisation;<br>RMO 4: Construction products regulation;<br>RMO 5: Providing information to consumers and retailers through labelling;<br>RMO 6: Voluntary industry agreement.	Adoption of RMO 1 with transitional period of 2 years as proposed by SEAC.

16	DecaBDE<br>(1163-19-5)	ECHA<br>(08/2014)	yes	The production volume reported for 2014 of 4000 t/y is considered to remain constant in the future. In addition, 400 t/y in articles are assumed to be imported to the EU	**RMO 1 (proposed and preferred option)**: Restriction on the manufacturing, use and placing on the market of decaBDE as a substance (in indoor/outdoor plastics and textiles), and decaBDE in mixtures and articles in concentrations ≥ 0.1 % by weight (exemption: Electrical and electronic equipment falling under the RoHS Directive);<br>RMO 2: Restriction of the manufacturing of decaBDE;<br>RMO 3: Restriction on the placing of the market of decaBDE;<br>RMO 4: Impose conditions on the waste management of decaBDE.	Adoption of RMO 1, with additional derogations as proposed by SEAC.
17	PFOA<br>(335-67-1)	German and Norwegian Competent Authorities<br>(10/2014)	yes	Current production volume of PFOA and its salts in EU approx. 1,250-12,000 t/y. Estimated volume after 2015 without restriction approx. 415-5,000 t/y	**RMO 1 (preferred option)**: Phase out of PFOA and PFOA-related substances as a substance, as constituents of another substance or in mixtures in concentrations equal or above 2ppm, within 18 months;<br>**RMO 2 (proposed option)**: Same as RMO 1 with possible exemptions.	Adoption of RMO 2 with derogations as suggested by RAC and SEAC, and with a deferral period of 3 years.
18	MeOH<br>(67-56-1)	Bureau for Chemical Substances Poland<br>(01/2015)	no	Current number of incidences (poisonings by ingestion of MeOH mixtures, mainly winter windshield washing fluids and denaturised alcohol containing high concentration of MeOH, 40-50% w/w) is considered to remain constant.	**Proposed restriction**: MeOH shall not be placed on the market for supply to the general public as a constituent of windshield washing fluids (including windshield defrosters) in concentration ≥ 3.0% by weight, and - as an additive to denaturated alcohol (*methylated spirit, denaturated alcohol, brennspiritus*) in concentrations ≥ 3.0% by weight	Adoption of the proposed restriction with stricter concentration limit (0.6% by weight) as proposed by SEAC.
19	D4/D5<br>(D4: 556-67-2<br>D5: 541-02-6)	Health and Safety Executive (HSE) UK<br>(06/2015)	yes	Current surface water emissions estimated at <5 t/y for D4 and <500 t/y for D5 (despite a high WWTP[e] removal efficiency). Releases are widely distributed in EU environment.	**RMO 1 (proposed and preferred option)**: Restriction on placing on the market and use of wash-off PCPs[g] containing > 0.1% w/w D4 or D5 with a compliance period of 2 years;<br>RMO 2: Restriction on placing on the market and use of wash-off PCPs containing > 0.1% w/w D4 or D5 with a compliance period of 5 years.	Adoption of RMO 1.
20	TDFA<br>(n.a.)	Danish Competent Authority for REACH, Danish Environment Protection	n.a.	Estimated reported poisoning incidents in EU28 are 330 - 660 incidents/y.	**RMO 1 (proposed and preferred option)**: Ban of mixtures containing TDFAs and organic solvent in spray products (aerosol dispensers, pump and trigger sprays and mixtures marketed for spray application) for consumer use in a concentration of TDFAs ≥ 2 ppb (w/w).	Adoption of RMO 1.

		Agency<br>(04/2016)			RMO 2: Risk-based ban of mixtures containing TDFAs and organic solvent in spray products for consumer use in a concentration of TDFAs ≥ 800 ppb. The TDFAs may be present as impurities.<br>RMO 3: Ban of mixtures containing TDFAs and organic solvent in aerosol dispensers for consumer use in a concentration of TDFAs ≥ 2 ppb (w/w).	
21	Phthalates (2017)<br>(DIBP 84-69-5<br>DBP 84-74-2<br>BBP 85-68-7<br>DEHP 117-81-7)	ECHA<br>(04/2016)	yes	Tonnages of DEHP, DBP, DIBP, and BBP estimated in articles placed on the EU28 market in the absence of a restriction is 171,000 t in 2014. Baseline projections are 121.000 t in 2020 and 143,000 t in 2039.	RMO 1: Restriction on the placement on the market of all articles containing the four phthalates DEHP, DBP, DIBP, and BBP;<br>**RMO 2 (proposed and preferred option)**: Restriction on the placement on the market of articles containing the four phthalates DEHP, DBP, DIBP, and BBP for: i) indoor use and ii) outdoor use, if there is a potential for contact with human skin or mucous membranes;<br>RMO 3: Proposed restriction without a derogation on food contact materials;<br>RMO 4: Restriction of the placing on the market of articles in the scope of the proposed restriction containing DEHP, DBP and DIBP only;<br>RMO 5: Proposed restriction with a derogation for DIBP in toys and childcare articles;<br>RMO 6: Restriction on the production as well as placing on the market of all articles.	The Commission considered the necessary conditions following Art. 68 of REACH not to be fulfilled. Restriction proposal not adopted.
22	Pb-PVC<br>(n.a.)	ECHA<br>(12/2016)	n.a.	Based on an estimate of 5.2 million workers exposed to isocyanates in all sectors (construction chemicals, automotive repair, other sectors), with a prevalence of 10%, the estimated number of new cases of occupational asthma in the EU is 6,500 persons/y.	**RMO 1 (proposed and preferred option)**: Implementation of restrictive conditions of use, mainly affected are workers who are at high risk, exemptions for workers at low risk;<br>RMO 2: Restrictive conditions of use are implemented to all workers who need to be trained without an option for exemption;<br>RMO 3: Complete ban of the use of diisocyanates and diisocyanates based products.	RAC/SEAC opinions adopted, Commission decision-process still on-going.
23	Diisocyanates<br>(n.a.)	Federal Agency for Occupational Safety (BAuA) Germany<br>(06/2017)	yes	In the absence of restriction measures, current (2016) levels of human (and environmental) Pb exposure from PVC applications in the EU are expected to remain unchanged. Estimated total tonnes of Pb in PVC articles between	**RMO 1 (proposed and preferred option)**: Restriction on lead and its compounds in all PVC articles with a concentration limit of 0.1% by weight of the PVC material of the article with derogations;<br>RMO 2: Restriction on Pb and its compounds in all PVC articles with a concentration limit of 0.1%, by	RAC/SEAC opinions adopted, Commission decision-process still on-going.

				1,057 – 4,263 t in 2016.	weight of the PVC material for all articles. No specific derogations;<br>RMO 3: Restriction on Pb and its compounds in all PVC articles with a concentration limit between 0.1 and 0.5% which will apply for all PVC articles (based on both virgin and PVC material), but with derogations.	
24	Pb shot (7439-92-1)	ECHA (03/2018)	yes	Several countries have no legislation to regulate the use of Pb gunshots in wetlands. Without a restriction 400,000 – 1,500,000 waterfowls are estimated to die annually in the EU from Pb poisoning. Of these, between 65,000 and 200,000 are estimated to occur in Member States without legislation prohibiting or reducing the use of Pb gunshot in wetlands.	**RMO 1 (proposed and preferred option)**: Restriction on the use of Pb gunshot in or over wetlands;<br>RMO 2: Restriction on the placing on the market and use of Pb gunshot;<br>RMO 3: Restriction on the use of Pb gunshot for all hunting;<br>RMO 4: Restriction on the use of Pb gunshot for all hunting of birds or hunting of waterfowl (e.g. ducks, geese and swans);<br>RMO 5: Restriction on the use of Pb gunshot in Ramsar Sites and/or SPAs in Natura 2000 network;<br>RMO 6: Phased approach to implementing a restriction on the use of Pb gunshot in wetlands;<br>RMO 7: No additional restrictions on the use of Pb gunshot.	RAC/SEAC opinions adopted, Commission decision-process still on-going.

Source: Information extracted from Annex XV Final Background Documents available at ECHA (2019d).

[†] Restriction dossiers for which opinions from the ECHA Risk Assessment and Socio-economic Assessment Committee (RAC and SEAC) have been adopted by December 2018.

[a] Lead in jewellery articles (Pb in jewellery); phenylmercury compounds (Phenyl-Hg); mercury in measuring devices (Hg); dibutyl phthalate (DBP), bis(2-ehtylhexyl)phthalate (DEHP), benzyl butyl phthalate (BBP), diisobutyl phthalate (DIBP) (phthalates 2012 and phthalates 2017); chromium VI compounds (CrVI); p-dichlorobenzene (DCB); lead in consumer articles (Pb cons. art.); nonylphenol varieties including ethoxylated nonylphenol (NP/NPE); *N*-methylpyrrolidone (NMP); cadmium and its compounds in paints and in artists' paints (Cd in paints and in artists' paints); chrysotile (CHRY); dimethylfumarate (DMFu); bisphenol-A (BPA); inorganic ammonium salts (NH_4); decabromodiphenyl ether (decaBDE); perfluorooctanoic acid, its salts and PFOA-related substances (PFOA); methanol (MeOH); octamethylcyclotetrasiloxane and decamethylcyclopentasiloxane (D4/D5); (3,3,4,4,5,5,6,6,7,7,8,8,8-tridecafluorooctyl) silanetriol and any of its mono-, di- or tri-O-(alkyl) derivatives (TDFA); lead compounds- polymers or copolymers of vinyl chloride (Pb-PVC); diisocyanates; and lead in shot (Pb shot).

[b] SVHC: Substances of Very High Concern. Definition according to dossier submitter.

[c] derived no-effect level (DNEL); [d] n.a.: Not applicable; [e] Waste water treatment plant (WWTP); [f] Bisphenol S (BPS); [g] Personal care products (PCPs).

Table S2: Regression results of a linear fitting of cost and benefit estimates presented in 13 REACH Annex XV restriction dossiers.

Model	Estimate	Standard error	t-value	p-value	Significance code	R^2
Linear regression						0.5768
Intercept: a	2.7516	1.2752	2.158	0.0539		
Log_{10} (costs*1e+06): b	0.7278	0.1880	3.872	0.0026	**	

Significance codes: 0 '***' 0.001 '**' 0.01 '*' 0.05 '.' 0.1 ' ' 1.
Source: Own calculations based on data presented in Table 3 (main manuscript).

Table S3: Discounting, time period and approach to uncertainty analysis in SEA of 24 REACH restriction dossiers[†]

No.	Substance[a]	Discounting approach and discount factor *r*	Time period of SEA[1]	Uncertainty analysis
1	Pb in jewellery	Exponential discounting, *r* = 3% applied to health impacts	Not specified	Discussion of uncertainties regarding the correlation between lead content and the lead migration rate.
2	Phenyl-Hg	Exponential discounting, *r* = 4% applied to costs of restriction options	2010-2028	Discussion of parameter uncertainties (related to emission estimates, cost estimates, import of articles, benefits of the restriction) and scenario uncertainty (definition of Phenyl-Hg decline in baseline scenario, future trends on export markets).<br>No quantitative uncertainty assessment.
3	Hg in measuring devices	Exponential discounting, *r* = 4% applied to costs of restriction options	20 years	Discussion of parameter uncertainty (release fractions, time period for SEA assessment, spill response, disposal of measuring devices, technical feasibility of alternatives).<br>Scenario analysis of compliance costs (high and low cost scenario) based on different estimates for mercury spill responses.
4	Phthalates (2012)	No discounting applied.	Not specified	Discussion of parameter uncertainty (exposure estimates, risk characterisation ratio, biomonitoring data, migration of phthalates in articles, number of articles, number of importers, substitution costs).<br>Sensitivity analysis of substitution costs (variation of phthalate content in articles and of expected outcomes of the authorisation process).
5	Cr VI	Exponential discounting of costs and benefits, *r* = 4%	20 years	Sensitivity analysis of cost and benefit estimates (variation of prevalence of chromium allergies, expected effect of the proposed restriction, welfare and industry costs). Additional sensitivity analyses provided by SEAC.
6	DCB	Exponential discounting of costs and benefits, *r* = 4%.	20 years	Sensitivity analysis (variation of assumed shape of demand curve (linear, non-linear), different price elasticities of demand, value of capital equipment regarding different discount rates).
7	Pb cons. art.	Exponential discounting of costs and benefits, with sensitivity analysis for assessing discounted lifetime productivity loss.	Lifetime	Discussion of legal and parameter uncertainties.<br>Scenario analysis of exposure scenario (revealing a high and low value of expected IQ loss), and of expected compliance costs and benefits (high, medium and low values of additional testing costs, substitution costs, costs for re-design, lead content in consumer articles, reduction of lifetime earnings due to IQ loss, discount rate applied to lifetime earnings).
8	NP/NPE	Exponential discounting of costs, r = 4%	2010-2021	Discussion of scenario and parameter uncertainty.<br>Scenario analysis of emission reduction capacity and cost estimates distinguishing high, mean and low parameter estimates.
9	NMP	Exponential discounting of costs, *r* = 4%.	15 years	Discussion of legal and parameter uncertainty (DALY[b] values, number of people exposed, cost estimates).<br>Sensitivity analysis (variation of number of workers exposed (values not published)).
10	Cd in paints	No discounting applied.	---	Discussion of uncertainty in estimates of Cd content.

11	Cd in artists' paints	Exponential discounting, using different discount rates for benefit (r = 3,5% and 2%) and cost estimates.	150 years	Explorative discussion of parameter uncertainty (Cd deposition, Cd concentrations in food categories, Cd concentrations in agricultural soils, effects of Cd exposure; scenario analysis of cost calculations (using different implementation time periods) and benefits (varying the values of the economic growth rate, the pure time preference rate, the length of the first generation in discounting, the direct cost growth rate and the monetary value per QALY[c]).
12	CHRY	Exponential discounting of costs and benefits, r = 4%.	2015-2025	Scenario analysis of costs (high and low cost estimates), assuming different rates of output and, production efficiency and time durability of the new technology.
13	DMFu	No discounting applied.	Not specified	Discussion of parameter uncertainty (number of people exposed, probability to suffer from dermatitis, costs of the use of an alternative substance, medical costs, loss of productivity, refund costs of articles).
14	BPA	Exponential discounting of benefits, using different discount factors for early and late benefits (r = 2% and 0%), exponential discounting of costs, r = 4%.	Health impacts: Lifetime with 2019 as reference year;<br>Costs: 2019-2030	Discussion of parameter and scenario uncertainty related to the assessment of health impacts (carcinogenicity effects, LOAEL[d], extrapolation of costs and benefit estimates to EU value of systemic bioavailability).<br>Sensitivity analysis of health impacts (variation of bioavailability factor, medical treatment costs, share of BPA containing thermal paper, share of cashiers) and costs (BPA concentration, BPA price, annual growth of thermal market).
15	NH_4	Exponential discounting of costs and benefits, r = 4%.	2017-2041	Discussion of parameter uncertainty (number of people exposed, volume and trends of export and import flows, health and environmental risks of alternative formulations).<br>Sensitivity analysis (variation of selected parameters of cost and benefit estimates).
16	DecaBDE	No discounting applied.	Different timescales for emissions.<br>Production: 2014<br>Article service life: 10 years<br>Waste landfills: 30 years	Discussion of parameter uncertainty (tonnage in imported articles, emissions, price difference between DecaBDE and alternatives, hazard profile of alternatives, trends of environmental concentrations).<br>Probabilistic assessment (Monte-Carlo analysis) of emissions to water.<br>Sensitivity analysis of substitution costs (variation of amount of DecaBDE used, emission factor, cost difference between DecaBDE and alternatives).
17	PFOA	No discounting applied.	Distinction between 'current' and 'post 2015' period	Discussion of parameter uncertainty (long-term exposure and effects, cost estimates, volumes of PFOA and PFOA-related substances, emissions, degradation rates).<br>Scenario analysis (worst-case and 'more realistic' estimates for volumes as drivers of cost estimates).
18	MeOH	No discounting applied.	Health impacts: Life year<br>Costs: 2004-2011	Discussion of parameter uncertainty (concentration of MeOH in windshield fluids, consumption of MeOH containing windshield fluids, costs of alternatives, number of people using MeOH as surrogate for consumable alcohol, medical care and treatment costs, costs of premature death, MeOH prices, MeOH tonnage).<br>Scenario analysis (high, central and low estimates of substitution costs and benefit estimates).
19	D4/D5	Not documented.	Distinction between a 2- and a 5-year transitional period	Discussion of parameter uncertainty (parameter values used for baseline cost assessment, expected product quality loss and corresponding WTP[e] values).<br>Qualitative uncertainty analysis (WWTP[f] release fraction to water).

				Sensitivity analysis (variation of tonnage values on release rates and emissions).
20	TDFA	Not documented.	Not specified.	Discussion of main uncertainties (number of poisoning incidents, total number of spray products, threshold concentration value).<br>Scenario analysis for consumer exposure, reformulation costs (conducted by SEAC).
21	Phthalates 2017	Exponential discounting of costs and benefits, *r* = 4%.	20 years	Discussion of parameter uncertainties (material costs, testing costs, substitution costs, costs of recycling, human health impacts) and scenario uncertainties (future tonnage, transition time to alternatives).<br>Scenario analysis (high and low tonnage value in baseline, high and low material and testing costs, high and low transition period on cost-effectiveness, impact of combined uncertainties on cost-effectiveness).<br>Sensitivity analysis (variation of infertility cases on health damage costs).
22	Diisocyanates	Exponential discounting of costs and benefits, *r* = 4%.	20 years	Discussion of parameter uncertainties (prevalence of asthma, age of asthma sufferers) and uncertainty due to lack of knowledge (other health effects except asthma; sensitivity analysis of cost estimates assuming high and low values for test exemption costs and training options.
23	Pb-PVC	Exponential discounting of benefits, *r* = 4%	Year of implementation (2016)	Sensitivity analysis of cost-effectiveness value, exploring the variation of the percentage of restriction-induced costs passed on to consumers on compliance costs and cost-effectiveness ratio.
24	PB in shot	Exponential discounting of replacement costs, *r* = 4%.	3 year transitional period<br>Replacement period shotguns 50 and 20 years, respectively	Discussion of parameter uncertainties (fraction of hunting taking place in wetlands, number of hunters involved in hunting on peatlands, number of guns to be replaced, lead emissions, annual number of waterbirds dying).<br>Scenario analysis (best, central and worst-case cost estimates and estimates of hunters affected).

Source: Information retrieved from Annex XV Final Background Documents available at ECHA (2019d).
[†] Restriction dossiers for which opinions from the ECHA Risk Assessment and Socio-economic Assessment Committee (RAC and SEAC) have been adopted by December 2018.
[a] Lead in jewellery articles (Pb in jewellery); phenylmercury compounds (Phenyl-Hg); mercury in measuring devices (Hg); dibutyl phthalate (DBP), bis(2-ehtylhexyl)phthalate (DEHP), benzyl butyl phthalate (BBP), diisobutyl phthalate (DIBP) (phthalates 2012 and phthalates 2017); chromium VI compounds (CrVI); p-dichlorobenzene (DCB); lead in consumer articles (Pb cons. art.); nonylphenol varieties including ethoxylated nonylphenol (NP/NPE); *N*-methylpyrrolidone (NMP); cadmium and its compounds in paints and in artists' paints (Cd in paints and in artists' paints); chrysotile (CHRY); dimethylfumarate (DMFu); bisphenol-A (BPA); inorganic ammonium salts (NH_4); decabromodiphenyl ether (decaBDE); perfluorooctanoic acid, its salts and PFOA-related substances (PFOA); methanol (MeOH); octamethylcyclotetrasiloxane and decamethylcyclopentasiloxane (D4/D5); (3,3,4,4,5,5,6,6,7,7,8,8,8-tridecafluorooctyl) silanetriol and any of its mono-, di- or tri-O-(alkyl) derivatives (TDFA); lead compounds- polymers or copolymers of vinyl chloride (Pb-PVC); diisocyanates; and lead in shot (Pb shot).
[b] Disability-adjusted life year (DALY), [c] Quality-adjusted life year (QALY), [d] Lowest observed adverse effect level (LOAEL), [e] Willingness to pay (WTP). [f] Waste water treatment plant (WWTP).
